# The humoral response to *Plasmodium falciparum *VarO rosetting variant and its association with protection against malaria in Beninese children

**DOI:** 10.1186/1475-2875-9-267

**Published:** 2010-10-05

**Authors:** Inès Vigan-Womas, Adjimon Lokossou, Micheline Guillotte, Alexandre Juillerat, Graham Bentley, André Garcia, Odile Mercereau-Puijalon, Florence Migot-Nabias

**Affiliations:** 1 Institut Pasteur, Unité d'Immunologie Moléculaire des Parasites, F-75015 Paris, France; 2 CNRS URA 2581, F-75015 Paris, France; 3 Institut de Recherche pour le Développement UMR216, Mère et enfant face aux infections tropicales, Paris, 75006, France; 4 Faculté de Pharmacie, Université Paris Descartes, Paris, 75270, France; 5 Institut des Sciences Biomédicales Appliquées, Cotonou, Benin; 6 Laboratoire de Parasitologie, Faculté des Sciences de la Santé, Cotonou, Benin; 7 Institut Pasteur, Unité d'Immunologie Structurale, 75015 Paris, France; 8 CNRS URA 2185, F-75015 Paris, France

## Abstract

**Background:**

The capacity of *Plasmodium falciparum*-infected erythrocytes to bind uninfected erythrocytes (rosetting) is associated with severe malaria in African children. Rosetting is mediated by a subset of the variant surface antigens PfEMP1 targeted by protective antibody responses. Analysis of the response to rosette-forming parasites and their PfEMP1 adhesive domains is essential for understanding the acquisition of protection against severe malaria. To this end, the antibody response to a rosetting variant was analysed in children recruited with severe or uncomplicated malaria or asymptomatic *P. falciparum *infection.

**Methods:**

Serum was collected from Beninese children with severe malaria, uncomplicated malaria or *P. falciparum *asymptomatic infection (N = 65, 37 and 52, respectively) and from immune adults (N = 30) living in the area. Infected erythrocyte surface-reactive IgG, rosette disrupting antibodies and IgG to the parasite crude extract were analysed using the single variant Palo Alto VarO-infected line. IgG, IgG1 and IgG3 to PfEMP1-varO-derived NTS-DBL1α_1_, CIDRγ and DBL2βC2 recombinant domains were analysed by ELISA. Antibody responses were compared in the clinical groups. Stability of the response was studied using a blood sampling collected 14 months later from asymptomatic children.

**Results:**

Seroprevalence of erythrocyte surface-reactive IgG was high in adults (100%) and asymptomatic children (92.3%) but low in children with severe or uncomplicated malaria (26.1% and 37.8%, respectively). The IgG, IgG1 and IgG3 antibody responses to the varO-derived PfEMP1 domains were significantly higher in asymptomatic children than in children with clinical malaria in a multivariate analysis correcting for age and parasite density at enrolment. They were essentially stable, although levels tended to decrease with time. VarO-surface reactivity correlated positively with IgG reactivity to the rosetting domain varO-NTS-DBL1α_1_. None of the children sera, including those with surface-reactive antibodies possessed anti-VarO-rosetting activity, and few adults had rosette-disrupting antibodies.

**Conclusions:**

Children with severe and uncomplicated malaria had similar responses. The higher prevalence and level of VarO-reactive antibodies in asymptomatic children compared to children with malaria is consistent with a protective role for anti-VarO antibodies against clinical falciparum malaria. The mechanism of such protection seems independent of rosette-disruption, suggesting that the cytophilic properties of antibodies come into play.

## Background

Despite recent scaling-up of control measures, *Plasmodium falciparum *malaria still claims about one million deaths each year, mainly young African children [1,2]. A hallmark of *P. falciparum *infection is the sequestration of infected erythrocytes (IE) in the microvasculature of vital organs [3-8] resulting from cytoadherence of mature IE to the endothelial cell lining and/or to other circulating cells or uninfected erythrocytes (rosetting) [9,10]. The *P. falciparum *Erythrocyte Membrane Protein 1 (PfEMP1), a variant adhesin displayed to the surface of the IE and encoded by the *var *gene family, plays a major role in IE cytoadherence [11-13].

There is a large body of evidence indicating that variant antigens dominate the response to the IE surface in children en route to acquiring protective immunity and that PfEMP1 molecules are major targets of the variant-specific responses [14-21]. The surface-exposed region of PfEMP1 has a modular structure with a succession of adhesion domains of two major types, namely the Duffy Binding-Like (DBL) domain and the cysteine-rich Inter-Domain Region (CIDR). Specific sequence signatures allow the classification of these adhesive domains in different classes (classes α, α_1_, β, γ, δ, ε, X for DBL; classes α, α_1_, β and γ for CIDR) [22]. Studies in endemic areas have shown that multiple DBL and CIDR domains elicit antibodies [19,23-25], but their association with protection remains unclear.

The rosetting and auto-agglutination cytoadherence phenotypes are consistently associated with severe malaria in African children [26,27]. Emerging evidence indicates that rosetting is mediated by proteins encoded by a subset of *var *genes, the exact number of which is still unknown. Three rosetting lines have been characterized, expressing respectively the FCR3S1.2/*IT4var21 *[28,29], the R29/*IT4var9 *[30] and the Palo Alto *varO *genes [31]. In all three lines the N-terminal DBL1α/α_1 _was identified as the binding domain for uninfected erythrocytes [28,30,31]. Little is known on the acquisition of antibodies to rosette-forming parasite types. In a pioneering study, Carlson *et al *reported that only 8% of children with cerebral malaria had antibodies disrupting the R+PAl rosettes (subsequently called FCR3S1.2) [28], compared to 38% in age-matched children with mild malaria [32], suggesting that rosette-disrupting antibodies contribute to protection against severe malaria. Whether antibodies to other rosetting types contribute to protection as well is unknown. It is not known either whether the antibody isotype, in particular cytophilic antibodies targeting the IE surface and promoting its opsonization also come into play to protect children against clinical malaria as observed in the *Saimiri sciureus *monkey [33,34]. Furthermore, how rosette-disrupting antibodies relate to IE-surface reacting antibodies and to their specificity with regard to the individual PfEMP1 domains exposed on the IE surface remains unclear.

Experimental obstacles to such studies have been the non-homogeneity of IE surface-displayed PfEMP1 molecules in cultivated lines (a consequence of antigenic variation) and the difficulty in producing appropriately folded recombinant PfEMP1 domains. A recombinant varO-NTS-DBL1α_1 _domain was produced in the native conformation that induced high titres of IE surface-reacting antibodies and rosette-disrupting antibodies [31,35]. Panning using a monoclonal antibody (mAb) raised to the recombinant domain allowed a "single variant" VarO culture to be established, in which all IE express the single VarO serotype [31]. A study in Senegal with this single variant culture showed that the seroprevalence of VarO was very high and strongly age-dependent, with high levels of VarO-IE surface-reactive and NTS-DBL1α_1_-reactive antibodies being reached at an age when effective protection against clinical malaria is established [31].

The present study was aimed at investigating seroprevalence to VarO-IE surface and to varO-PfEMP1 recombinant domains in a second, geographically distant endemic area exposed to different transmission conditions and at analysing its possible association with protection against severe and/or uncomplicated clinical malaria. The antibody response to the VarO serotype was investigated in Benin among immune adults, semi-immune children with *P. falciparum *asymptomatic parasitaemia and children with mild or severe *P. falciparum *malaria disease. The varO-IE surface seroreactivity and VarO rosette disrupting capacity were analysed. Total IgG, IgG1 and IgG3 responses to three PfEMP1-varO recombinant domains (NTS-DBL1α_1_, CIDR1γ and DBL2βC2), which could be produced as soluble, properly folded recombinant proteins capable of inducing high titres of VarO surface-reacting antibodies in the mouse, were assessed. Data indicate an elevated seroprevalence to varO-IE surface and recombinant domains in asymptomatic children and immune adults, contrasting with a low prevalence and low antibody levels in children with clinical malaria. Intriguingly rosette-disrupting antibodies were rare in immune adults and not detectable in all children including in asymptomatic children, suggesting that mechanisms other than prevention of rosetting may operate to protect against VarO parasites.

## Methods

### Study areas and sample collection

Clinical and parasitological characteristics of the recruited subjects are presented in Table 1. From July 2006 to January 2007, 102 children with symptomatic *P. falciparum *malaria were recruited in three health centres (Saint-Luc, Béthesda and Cotonou hospitals) located in the urban area of Cotonou where malaria transmission is perennial, with two seasonal peaks corresponding to rainy seasons, from April to July and September to November. A survey conducted in 2000 showed heterogeneous malaria transmission in the city of Cotonou, with transmission varying from 5, 29 and 47 infective bites per person per year near the beach, in the centre of the city and in the outer-urban lagoon areas, respectively [36]. The inoculation rates were undoubtedly lower during the study period, due to the introduction of effective prevention and therapy. According to clinical and parasitological features at admission described elsewhere [37], children were assigned to the severe malaria (SM, n = 65) or uncomplicated malaria (UM, n = 37) group. SM was defined as the association of fever (axillary temperature ≥ 37.5°C), presence of circulating *P. falciparum *ring forms and a neurologic Blantyre score < 3. Forty-two SM children presented cerebral malaria (coma duration ≥ 6 h) and eight had severe malarial anaemia (haemoglobin level < 5 g/dL). The fifteen remaining SM children combined a Blantyre coma score < 3, coma duration < 6 h as well as haemoglobin level ≥ 5 g/dL but clinical signs of anaemia. For the UM group, inclusion criteria were fever or history of fever during the last 24 hours, the presence of circulating *P. falciparum *ring forms and a Blantyre coma score ≥ 3. Blood was withdrawn before the administration of any drug. An anti-malarial treatment was administered in accordance with the treatment practices of each hospital.

**Table 1:** Clinical and parasitological characteristics of the children at enrolment (year 2006): *P. falciparum *asymptomatic children (AP), children with uncomplicated malaria (UM) and children with severe malaria (SM).

Parameters	AP (n = 52)	UM (n = 37)	SM (n = 65)	*P*
Sex ratio (M/F)	0.9 (24/28)	1.8 (24/13)	0.8 (29/36)	0.11 ^b^
Age (yrs) mean ± SD	6.5 ± 1.3	4.7 ± 3.4	2.9 ± 1.8	AP > UM > SM (*P *< 0.0001) ^c^
Parasite density (/μl) ^a^	811 (369-1,751)	24,900 (3,606-65,440)	61,920 (1,684-192,000)	AP < UM/SM (*P *< 0.001) ^d^

^a ^Median parasite density (interquartile range IQ25-75).

^b ^*P *value of the χ^2 ^test.

^c ^*P *value of the Student's unpaired-*t*-test.

^d ^*P *value of the Mann-Whitney *U*-test.

In December 2006, 52 children with asymptomatic falciparum parasitaemia (AP) were enrolled in the primary schools of Ouidah, a semi-rural town situated 35 km west from Cotonou. The entomological inoculation rate averaged 2.05 ± 1.28 infective bites per human per 100 nights in the nearby rural area of Tori Bossito [38]. AP children were non-febrile at enrolment or during the preceding 24 hours, but presented circulating *P. falciparum *ring forms. In two out of 52 cases, the appearance of fever in a delay of 3 days after enrolment led to the administration of antipyretics associated with an artemisinin-based combination therapy (ACT), as recommended by the National Malaria Control Programme. Another blood sample was collected from the same children one year later (January 2008).

Plasma samples from 30 healthy adults (HA) living in the rural area of Tori-Bossito, which is located halfway of Cotonou and Ouidah were used as controls of the acquired anti-malarial immunity in this area. These adults were bled in November 2008.

For each individual, a venous blood sample was collected in EDTA Vacutainer tubes. Plasmas were collected and stored at -20°C for subsequent antibody testing. Blood smears were prepared to determine blood-stage infection and parasitaemia by microscopy. Negative control plasmas were obtained from 20 healthy Europeans adults who had not been exposed to malaria (Blood bank, EFS-Rungis, France) and were used either individually or grouped into a negative control pool (NC). For a positive control (PC), a pool of plasma taken from adult donors living in Dielmo, Senegal, was used, that showed high antibody reactivity to the surface of VarO-IE [31].

This study has been approved by the ethic committee of "Faculté des Sciences de la Santé" of the University of Abomey-Calavi in Benin. For each child, a written informed consent from parents or legal guardians was obtained. The study was conducted in accordance with the Declaration of Helsinki.

### Surface immunofluorescence assay (S-IFA) and rosette disruption

Single variant Palo Alto 89F5 VarO parasites were cultivated in human O^+ ^RBC. Weekly enrichment of rosettes, monthly positive selection by panning on a mouse monoclonal antibody raised to the varO-NTS-DBL1α_1 _domain were done as described [31]. For surface immunofluorescence assay, an aliquot of rosette-enriched 89F5 VarO parasites was incubated with the plasma sample (final dilution 1:20). Total IgG binding was detected as described [31]. Surface reactivity was expressed in arbitrary units (AU) as follows: [(% IE^+^_sample _- % IE^+^_NC_)/(% IE^+^_PC _- % IE^+^_NC_)] × 100. The mean surface reactivity plus 3 standard deviations observed with plasma samples from 20 non-immune malaria individuals was used to set the positivity threshold (20 AU).

For the rosette disruption assay, an aliquot of VarO rosette-enriched parasites (20 μL, 5% parasitaemia, > 85% rosetting frequency, in complete RPMI culture medium) was incubated with plasma (final dilution 1/5) for 30 min at 37°C as described [31]. Each plasma sample was assayed in duplicate. The rosetting rate was compared to a control culture in complete RPMI medium in presence of the NC pool. The positive control was PC, a pool of hyper-immune sera of adults from Dielmo (Senegal) and able to disrupt VarO rosettes [31].

### Recombinant PfEMP1-varO domains

The soluble recombinant NTS-DBL1α_1 _domain (residues 1-487 of the predicted varO amino-acid sequence, GenBank accession number EU908205) was produced in insect cells [31]. Construction of the recodoned recombinant varO-CIDRγ (residues 508 to 787) and varO-DBL2βC2 (residues 831 to 1241) domains and production of recombinant proteins will be described in detail elsewhere (Guillotte *et al*, in preparation). Briefly, both domains were produced from a recodoned coding sequence where all potential N-glycosylation sites were mutated. VarO-DBL2βC2 was produced in the baculovirus/insect cells system whereas varO-CIDRγ was produced in *Pichia pastoris*. Each domain was produced as a soluble protein with a C-terminal hexa-histidine-tag. Protein purity was evaluated by sodium dodecyl sulfate-polyacrylamide gel electrophoresis (SDS-PAGE) and Western blot. The protein sequence was verified by N-terminal sequencing and mass spectrometry analysis. Alike the NTS-DBL1α_1 _domain [31,35] the recombinant varO-CIDRγ and varO-DBL2βC2 induced varO-IE surface reactive antibodies in the mouse (Guillotte *et al*, in preparation). The other varO-derived DBL domains (DBL3, 4 and 5) were not available as recombinant proteins with native folding and were not used in this study.

### Enzyme-linked immunosorbent assay (ELISA)

A crude extract of VarO-IE was prepared as described [39]. IgG levels were quantified in plasma diluted 1/100 essentially by ELISA as described [31] using i) 100 μL of the varO-IE crude extract or ii) 0.2 μg of recombinant protein. For total immunoglobulin-G (IgG) detection, plates were probed with an horseradish peroxidase (HRP)-conjugate goat anti-human IgG-F(ab')2 (Cappel, France; dilution 1/7,500) [31]. For IgG-subclass (IgG1 and IgG3) analysis, plates were first incubated with a mouse anti-IgG1 (clone NL16; dilution 1/2,000; Skybio, England) or anti-IgG3 (clone ZG4; dilution 1/10,000; Skybio, England) and probed with an HRP-conjugate goat anti-mouse IgG (Promega, France; dilution 1/3,000). Each serum was tested in duplicate. Negative (NC) and positive (PC) controls were included in each plate. Results were expressed in arbitrary units (AU) calculated with the formula: 100 × [ln(OD _tested plasma_) - ln(OD _NC_)]/[ln(OD _PC_) - ln(OD _NC_)] [40]. The threshold for positivity was determined for each antigen and each class/subclass from the 95^th ^percentile of the antibody reactivity of 20 individual plasma samples from non-immune malaria individuals. For varO-NTS-DBL1α_1 _it was 43.2, 14.0 and 17.3 AU for total IgG, IgG1 and IgG3 respectively; for varO-CIDRγ it was 30.6, 47.1 and 40.5 for total IgG, IgG1 and IgG3, respectively and for varO-DBL2βC2 it was 32.4, 70.3 and 39.6 for total IgG, IgG1 and IgG3, respectively. The positivity threshold was set at 9.0 AU for total IgG to VarO-IE crude extract.

### Statistical analysis

Differences in proportions were analysed using the χ^2 ^test. Differences in means were tested by the non-parametric Mann-Whitney *U*-test, except for age, where the Student's unpaired *t*-test was employed as age was normally distributed. Statview 5.0 (SAS Institute Inc., Cary, NC) was used for these calculations. The associations between antibody responses and covariates (sex, age, *P. falciparum *carriage at blood drawing) found to be significant in the univariate analysis were investigated by multiple linear regression analysis using STATA (StataCorp. Release 8.0). The antibody levels in 2006 and 2008 for children sampled at these 2 time-points (n = 45) were compared using a non-parametric Mann-Whitney *U*-test. In case of *P *value less than 0.20 in the univariate analysis, a multiple linear regression was performed including age as covariate, to take into account the effect of aging on the evolution of antibody levels. For all tests, *P *values of less than 0.05 were considered significant.

## Results

### Characteristics of the subjects recruited

Table 1 summarizes the main characteristics of the three groups of children recruited for this study. *P. falciparum *asymptomatic children (AP, n = 52) were older than children with uncomplicated malaria (UM, n = 37) and children with severe malaria (SM, n = 65) (Student's *t*-test, *P *< 0.0001, for comparison between the groups). Asymptomatic children presented a lower median parasite density than children with uncomplicated or severe malaria (*P *< 0.001). There was no difference regarding gender, age and parasite density between SM clinical sub-groups. The control group of immune adults (HA, n = 30) comprised 15 men and 15 women, older than 20 years, who were healthy at the time of blood drawing.

### VarO-IE surface-reactive antibodies: seroprevalence and levels

**Figure 1:**
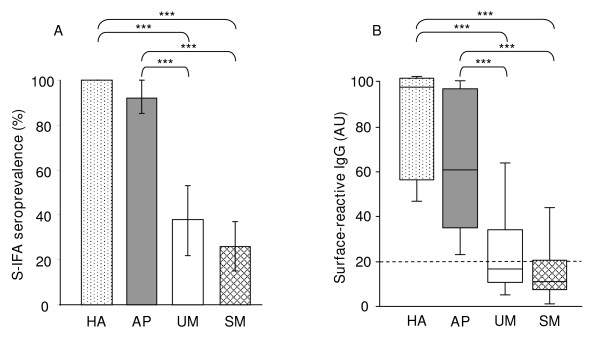
**Surface-reactive antibodies to single-variant culture of VarO-IE**. Plasma samples were obtained from healthy adults (HA, n = 30) and children with asymptomatic *P. falciparum *infection (AP, n = 52), uncomplicated (UM, n = 37) or severe (SM, n = 65) malaria. For each child, antibodies (total IgG) reacting with the surface of live VarO-IE were analyzed by S-IFA and flow cytometry. Results are expressed in arbitrary units (AU, see Materials and Methods). (A) Prevalence (95% CI) of surface-reactive antibodies to VarO parasites in the four groups of individuals: HA, AP, UM or SM. (B) Surface reactivity levels in HA, AP, UM or SM children. Box-whisker plots illustrate medians with 25^th ^and 75^th ^percentiles, and whiskers for 10^th ^and 90^th ^percentiles. The outlying dots indicate values exceeding the 90^th ^and 10^th ^percentiles. The *P *values were estimated using the χ^2 ^test (A) and the nonparametric Man-Whitney *U-*test (B). ***, *P *< 0.0001.

Antibodies (total IgG) reacting with the VarO-IE surface were studied using a single-variant parasite culture with more than 95% IE at mature stages expressing the PfEMP1-varO adhesin. All healthy, immune adults reacted against the VarO-IE surface. Seroprevalence rate was higher in AP children (92.3%, 95% confidence interval [CI], 85.1 to 99.6) than in UM and SM children (37.8% [95% CI, 22.2 to 53.5] and 26.2% [95% CI, 15.5 to 37.8], respectively) (Figure 1A). This difference between AP children and children with clinical malaria was highly significant (χ^2 ^test, *P *< 0001), but there was no statistical difference between UM and SM children as well as between UM and the CM sub-group of SM children.

The level of VarO-IE surface reactive antibodies differed in the three groups of children, from nil or very low in SM (median 11.21 AU, interquartile range IQ25-75 = 7.57 - 20.25 AU), to low in UM children (median 16.71 AU, IQ25-75 = 10.85 - 33.67 AU) and high in AP children (median 61.35, IQ25-75 = 35.19 - 97.5 AU). The highest levels were observed for HA adults (median 97.71, IQ25-75 = 57.54 - 101.76 AU) (Figure 1B). The surface-reactive antibody levels did not differ between UM and SM (whole group but also CM sub-group), but differed significantly between children with clinical malaria and AP children. Multivariate analysis conducted for children only confirmed the clinical malaria-dependence of the level of VarO-surface reactive antibodies (-17.07, *P *< 0.0001) in spite of a small positive effect of age on antibody levels (1.91, *P *= 0.052), the parasite density being without effect on these observations (*P *= 0.12).

**Figure 2:**
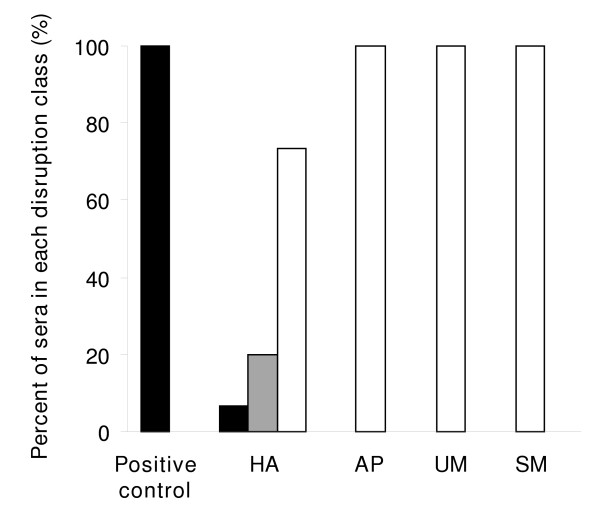
**Percentages of plasma samples with an anti-varO rosetting activity more than 50% (black), from 10 to 50% (grey) and below 10% (white), in positive controls, HA, SM, UM and AP groups**.

The VarO-rosette dissociation assay showed that few HA sera displayed anti-varO rosetting activity. Only 2 of 30 sera disrupted > 50% of the VarO rosettes of the culture, and 6 disrupted 10-50% of the rosettes. Most sera had marginal to nil rosette disrupting activity, contrasting with sera from Senegalese adults (Figure 2). None of the children sera harbouring surface-reactive antibodies (be they from asymptomatic or symptomatic children) had detectable antibodies able to disrupt VarO rosettes.

### Seroprevalence to VarO-IE crude extract and varO recombinant domains

Total IgG, IgG1 and IgG3 responses to the individual varO recombinant domains were analysed by ELISA. The sandwich ELISAs for IgG1 and IgG3 detected specific responses in some children scored as negative for total IgG reactivity, likely reflecting their higher sensitivity. To rule out a technical issue with the quality of the sera, total IgG reactivity with the VarO-IE crude extract was determined.

**Figure 3:**
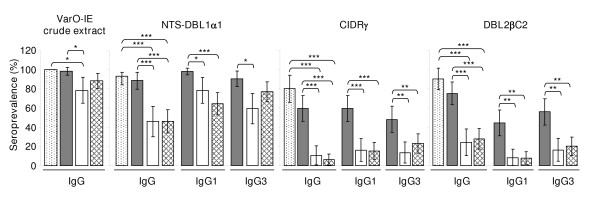
**Prevalence rates of IgG to VarO-IE crude extract, and of IgG, IgG1 and IgG3 to varO recombinant NTS-DBL1α_1_, CIDRγ and DBL2βC2 in healthy adults (dots) and the children recruited in 2006, AP (grey), UM (white), SM (criss-cross)**. Bars denote the 95% confidence interval. Asterisks indicate *P *values estimated using the χ^2 ^test as follows: ***, *P *< 0.0001; **, *P *< 0.001; *, *P *< 0.01.

In none of the assays did UM and SM children, as well as UM and CM children, display a statistically different response (Figure 3). Total IgG seroprevalence to the recombinant antigens and the crude extract was similarly high in HA adults and AP children. Seroprevalence to the crude extract was lower in UM children. Total IgG and IgG1 seroprevalence to the three recombinant domains was higher in AP children than in UM and/or SM children. Similar results were observed for IgG3 except for seroprevalence to NTS-DBL1α_1 _which was higher in AP children than UM children but not different from SM children (Figure 3).

NTS-DBL1α_1 _was the most frequently recognized domain. For total IgG, the prevalence rates among children were NTS-DBL1α_1 _> DBL2βC2 > CIDRγ (paired comparisons analysed by χ^2^, all significant: odds-ratio (OR) NTS-DBL1α_1_/DBL2βC2 = 7.7 [95% CI, 3.5 to 17.1]; NTS-DBL1α_1_/CIDRγ = 19.5 [95% CI, 4.5 to 84.7]; DBL2βC2/CIDRγ = 8.9 [95% CI, 3.7 to 21.2]; *P *< 0.0001 for each). Similar results were obtained for IgG1 and IgG3 seroprevalence rates.

**Figure 4:**
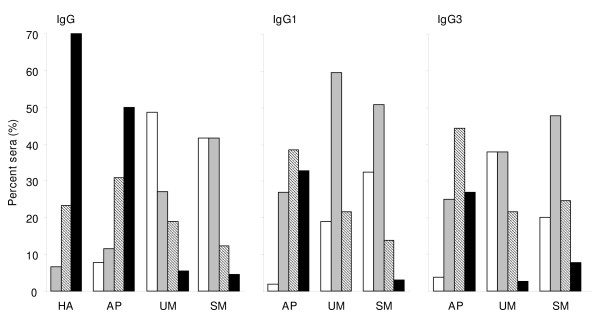
**Frequency distribution of the reactivity to one or more PfEMP1-varO domain as by groups of healthy adults (HA) and children recruited in 2006 (AP, UM or SM as indicated) and by Ig assay as indicated (total IgG for HA; total IgG, IgG1 and IgG3 for AP, UM and SM)**. Reactivity was assessed by ELISA on individual domain as indicated in the Methods section. A plasma sample was classified as seropositive when the recorded signal was above the threshold. Seroreactivity to 1, 2 or 3 domains is indicated irrespective of the actual domain recognized in each class. Symbols used for bars: no domain recognized (white), any one domain recognized (grey), two domains recognized (hatched), all three domains recognized (black).

When examining the number of individual varO-derived recombinant domains recognized in each group, it was clear that HA adults had a broad reactivity, with most sera (70%) reacting with each of the three domains (Figure 4). The response in AP children was narrower, as about 50% and 30% had antibodies reacting with three or two domains, respectively. In contrast, the IgG response of children with clinical malaria (UM or SM) was quite restricted, with 49% and 42% respectively with no detected seroreactivity to any of the three antigens (Figure 4). The more sensitive IgG1 and IgG3 assays reduced the percentage of seronegative children in each clinical group, but still outlined a more restricted response of the UM and SM children compared to AP children (for each Ig assay, *P *< 0.0001 for AP *vs*. UM and AP *vs*. SM when comparing reactivity to 0-1 and 2-3 domains).

### Antibody levels to VarO-IE crude extract and varO recombinant domains

HA adults and AP children presented similar total IgG levels, except for VarO-IE crude extract (*P *= 0.0004) (Table 2). NTS-DBL1α_1 _was the only recombinant domain to generate lower total IgG, IgG1 and IgG3 levels in UM than in AP children (*P *= 0.02, *P *< 0.0001 and *P *< 0.0001, respectively). SM children had lower total IgG, IgG1 and IgG3 levels to NTS-DBL1α_1 _than AP children (*P *= 0.002, *P *< 0.0001 and *P *= 0.01, respectively), as well as lower total IgG and IgG1 to CIDRγ (*P *= 0.04 and *P *= 0.007, respectively). The antibody levels did not significantly differ in children with UM and SM, except total IgG to VarO-IE crude extract (*P *= 0.03) and IgG3 to NTS-DBL1α_1 _(*P *= 0.007) (Table 2). Similar differences were observed when considering the CM sub-group of SM children, except for CIDRγ which elicited comparable antibody levels among HA, AP, UM and CM groups.

**Table 2:** Median values of antibody responses to varO-IE crude extract and varO recombinant domains, for healthy adults (HA) and children (AP, UM and SM, in 2006)

Antigens	Median values (AU) ^a^			
	HA (n = 30)	AP (n = 52)	UM (n = 37)	SM (n = 65)
**varO-IEcrude extract IgG**	95.6 (90.2-97.1)	86.6 (78.4-93.5) ^b^	86.4 (69.7-90.1) ^c^	72.2 (61.1-84.2)^d, f, g^
**NTS-DBL1α_1 _:**				
**IgG**	93.7 (88.9-99.5)	92.3 (74.0-105.9)	65.7 (61.9-79.9) ^c, e^	64.4 (52.5-83.8) ^d, f^
**IgG1**	nd	93.3 (82.2-99.3)	34.7 (21.6-50.1) ^e^	37.0 (29.9-60.3) ^f^
**IgG3**	nd	64.3 (42.9-80.2)	35.7 (27.4-46.8) ^e^	46.3 (36.3-64.6) ^f, g^
**CIDRγ:**				
**IgG**	88.9 (63.6-103.4)	64.2 (49.7-100.9)	74.4 (53.3-107.3)	47.2 (39.3-52.4) ^d, f^
**IgG1**	nd	83.5 (65.9-97.4)	75.1 (55.4-95.7)	65.1 (51.2-68.9) ^f^
**IgG3**	nd	65.7 (51.4-81.0)	94.0 (64.2-107.0)	63.9 (45.7-70.0)
**DL2βC2:**				
**IgG**	73.2 (59.4-90.4)	68.7 (47.0-82.2)	54.8 (43.4-70.8)	47.8 (37.6-68.3) ^d^
**IgG1**	nd	93.3 (78.8-101.0)	82.8 (75.6-93.0)	81.9 (76.9-93.2)
**IgG3**	nd	64.2 (47.6-77.0)	48.2 (44.0-53.8)	69.8 (50.7-88.6)

nd: not done

^a ^Median values (25th-75th percentiles), including responders only, and expressed in arbitrary units (AU).

Significant differences (Mann-Whitney *U*-test, *P *< 0.05) between median values of HA and AP responders (^b^), HA and UM responders (^c^), HA and SM responders (^d^), AP and UM responders (^e^), AP and SM responders (^f^), and UM and SM responders (^g^).

Importantly, age and parasite density were not associated with any of the antibody level assayed in each of the three groups of children, although this should be qualified because of the limited number of responders. A multivariate analysis taking into account sex, age and parasite density confirmed higher levels of total IgG and IgG subclasses in AP compared to symptomatic children (all antigens *P *< 0.02, except for total IgG to NTS-DBL1α_1 _[*P *= 0.05] and to DBL2βC2 [*P *= 0.06]). The observed differences did not correlate with sex and parasite density, and in some cases, the presence of an age-related increase in antibody (IgG to NTS-DBL1α_1 _[*P *= 0.001] and to DBL2βC2 [*P *< 0.0001] as well as IgG1 to DBL2βC2 [*P *< 0.0001]) did not counterbalance the strong impact of the clinical presentation.

In each clinical group, the total IgG and IgG1 levels reactive to an individual antigen were positively correlated (Table 3). Total IgG and IgG1 were moderately related to the IgG3 levels for NTS-DBL1α_1 _(Rho from 0.39 to 0.59, all *P *< 0.02) and for CIDRγ (Rho from 0.38 to 0.49, all *P *< 0.003), and somewhat stronger with DBL2βC2 (Rho from 0.42 to 0.72, all *P *< 0.002) (Table 3). Results were the same when considering the CM sub-group, except for total IgG levels directed to CIDRγ which were not related to IgG1 and IgG3 levels (Rho = 0.20 and 0.26; *P *= 0.20 and 0.10, respectively).

**Table 3:** Correlations between IgG, IgG1 and IgG3 levels to varO recombinant domains, in children from AP, UM and SM groups (in 2006)

Correlations	NTS-DBL1α_1_	CIDRγ	DL2βC2
**IgG *vs*. IgG1:**			
**AP (n = 52)**	0.64; *P *< 0.0001 ^a^	0.87; *P *< 0.0001	0.79; *P *< 0.0001
**UM (n = 37)**	0.42; *P *= 0.01	0.72; *P *< 0.0001	0.81; *P *< 0.0001
**SM (n = 65)**	0.68; *P *< 0.0001	0.49; *P *< 0.0001	0.83; *P *< 0.0001
**IgG *vs*. IgG3:**			
**AP**	0.45; *P *= 0.0008	0.45; *P *= 0.0008	0.42; *P *= 0.002
**UM**	0.39; *P *= 0.02	0.49; *P *= 0.002	0.72; *P *< 0.0001
**SM**	0.55; *P *< 0.0001	0.38; *P *= 0.002	0.64; *P *< 0.0001
**IgG1 *vs*. IgG3:**			
**AP**	0.44; *P *= 0.0009	0.44; *P *= 0.001	0.56; *P *< 0.0001
**UM**	0.59; *P *= 0.0001	0.48; *P *= 0.003	0.71; *P *< 0.0001
**SM**	0.44; *P *= 0.0002	0.44; *P *= 0.0002	0.57; *P *< 0.0001

^a ^Spearman's rank correlation test (Rho; *P*)

Positive correlations between domain-reactive antibodies were observed in the AP group for total IgG (NTS-DBL1α_1 _*vs*. CIDRγ: Rho = 0.63, *P *< 0.0001; NTS-DBL1α_1 _*vs*. DBL2βC2: Rho = 0.49, *P *= 0.0002 and CIDRγ *vs*. DBL2βC2: Rho = 0.53, *P *< 0.0001) and IgG1 (NTS-DBL1α_1 _*vs*. CIDRγ: Rho = 0.40, *P *= 0.004; NTS-DBL1α_1 _*vs*. DBL2βC2: Rho = 0.46, *P *= 0.0006 and CIDRγ *vs*. DBL2βC2: Rho = 0.40, *P *= 0.003). In the UM and SM groups, a strong correlation was observed between total IgG levels to NTS-DBL1α_1 _and DBL2βC2 (Rho = 0.74 and 0.52, *P *< 0.0001, respectively) but not between the other domains, whether for total IgG or for IgG1 and IgG3. The same profile of relationships was confirmed for the CM sub-group of SM children.

### Relationships between surface-reactive, varO-IE extract-reactive and recombinant domain-reactive antibodies

**Figure 5:**
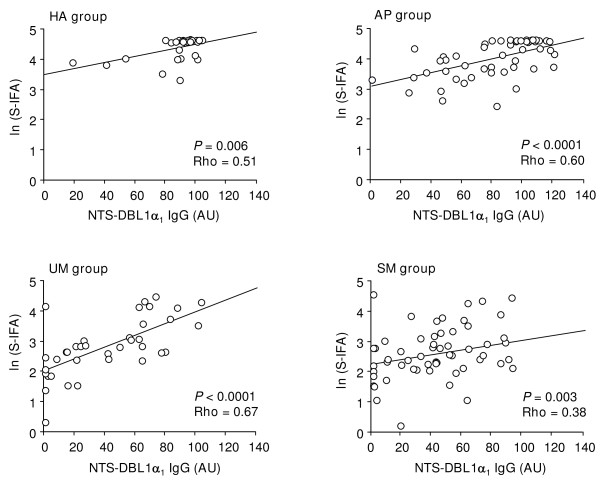
**Correlation between S-IFA and antibody-reactivity to the NTS-DBL1α_1 _protein: linear regression of ln(S-IFA) [ordinate] as a function of NTS-DBL1α_1 _IgG levels (AU) [abscissa] in HA, AP, UM and SM groups**.

For all four groups, the strongest correlations involving VarO-surface reactive antibodies were observed with total IgG to NTS-DBL1α_1 _(Rho from 0.38 to 0.67, all *P *< 0.006) (Figure 5). A similar observation was valuable for the CM sub-group (Rho = 0.37, *P *= 0.02). Less numerous and strong correlations were observed between VarO-surface reactive antibodies and IgG1 or IgG3 levels to varO-domains. Antibodies to the VarO-IE crude extract correlated to none of the antibody level to the individual varO-domains in the HA and AP groups (all Rho < 0.06, all *P *> 0.18).

### Temporal evolution of VarO seroreactivity in asymptomatic *P. falciparum *carriers

Forty-five out of the 52 children with asymptomatic *P. falciparum *infection in December 2006, were bled again in January 2008 in order to follow the temporal evolution of their specific antibody response. Among them, six were parasitaemic for *P. falciparum *in 2008, with a low median parasite density (78 ring forms per microliter of blood, IQ25-75 = 36-158), i.e. were asymptomatic parasite carriers. The prevalence rate of VarO-IE surface IgG was lower in 2008 than in 2006 but not that of VarO-IE crude extract IgG (additional file 1). A similar decrease between 2006 and 2008 was observed for the prevalence rates of total IgG, IgG1 and IgG3 to the three recombinant proteins, except for IgG1 to NTS-DBL1α_1 _as well as total IgG and IgG1 to CIDRγ, which remained unchanged (additional file 1). Levels of antibodies to the VarO-IE crude extract dropped in 2008 (*P *= 0.0007) whereas antibodies reacting with the other antigens remained stable (additional file 1).

Multivariate analysis confirmed the negative effect of time, consistent with a diminution between 2006 and 2008 of VarO-IE crude extract antibodies (-18.0, *P *< 0.0001) independently of a positive effect of age (3.4, *P *= 0.04) reflecting the ongoing acquisition if immunity. A similar observation was made for IgG1 to NTS-DBL1α_1 _with a negative effect of time (-10.0, *P *= 0.04) dominating the positive effect of age (4.2, *P *= 0.03).

## Discussion

Although rosetting is the best-documented cytoadherence phenotype associated with severe malaria in African children, little is known on the response acquired to rosetting parasites in endemic areas. The present study in Benin confirms the elevated seroprevalence of VarO in semi-immune children and immune adults observed in a Senegalese population [31]. Children with severe or uncomplicated malaria had a much lower anti-varO response than semi-immune asymptomatic children, consistent with the conclusion that these antibodies are associated with protection against clinical malaria in the age group that progressively mounts a protective response.

A very high prevalence of the antibodies reacting with the VarO-IE surface was observed in AP children. The mean age of the AP children was 6.5 ± 1.3 y, an age at which children living under such transmission conditions are semi-immune but are still at risk in developing clinical malaria. As such, and with the caveats of comparisons between different studies, the seroprevalence to VarO-IE in AP children from Ouidah seems higher than the response to other parasite lines, including the rosette-forming FCR3S1.2 parasites [41] or a single variant A4var line observed in semi-immune Kenyan children living under similar transmission conditions [20], or to the response to local isolates reported in Tanzanian children living in low and moderate transmission conditions [25]. It is also higher than the response observed against a panel of local isolates in Ghanaian children living in more intense transmission conditions who had supposedly acquired earlier in life an expanded antibody repertoire [21]. While these data require further confirmation, they are consistent with VarO being a so-called "frequent" or "prevalent" serotype [42,43] usually associated with severe malaria, which seems to be a feature of group A *var *genes [23,44-47] to which the *varO *gene belongs. It is not known at present whether the reaction observed with the VarO-IE surface and/or the various -*varO*-derived recombinant domains is strictly *varO*-specific or reflects a broad cross-reactivity to other "rosetting variants" some of which also belong to group A *var *genes [46,48].

Prevalence rates and levels in all VarO-related assays (IE surface, total IgG, IgG1 and IgG3 to the three recombinant domains) were much lower in the children with clinical malaria than in AP children. This difference remained significant in the multivariate analysis, i.e. after correcting for age and parasite density at enrolment. It may be possible that the different exposure of children partly contribute to these findings. However, AP children were recruited in an area where transmission intensity was estimated to be lower than in the Cotonou area where symptomatic children recruited at hospital lived, although transmission in Cotonou is quite heterogeneous. Be that as it may, this difference would translate into a delayed acquisition of antibodies in Ouidah compared to Cotonou, as higher transmission intensity is clearly associated with a more rapid acquisition of an expanded antibody repertoire [23-25]. To further document the association of VarO-reacting antibodies with protection against clinical malaria, a longitudinal follow-up of children is needed to show that the presence of such antibodies prevents disease caused by *P. falciparum *parasites expressing this serotype or cross-reacting serotypes.

Of the three recombinant domains studied here, NTS-DBL1α_1 _had the highest seroprevalence and the highest antibody levels. In view of the known mosaic structure of the *var *genes, this probably indicates that parasites expressing the varO-NTS-DBL1α_1 _domain or a related cross-reacting domain may not co-express varO-CIDRγ-like or varO-DBL2βC2-like domains. Each domain elicits antibody in the context of where it is presented, i.e., not necessarily associated with the same domains as those in the *varO *gene. This might account for the limited correlation of the response against the individual domains in the children with clinical malaria. Although it is tempting to speculate that the higher seroreactivity to varO-NTS-DBL1α_1 _may reflect the generally high conservation of DBL1α_1 _sequences across the *var *repertoire and between isolates compared to CIDRγ, for example, which are quite diverse, further study is needed to test this hypothesis. Furthermore, comparison of reactivity between different antigens must be interpreted with caution because detection depends on the sensitivity of the assay, which may vary from one to the other, and because it is difficult to compare arbitrary units. Notwithstanding these reservations, the observation of a higher response to NTS-DBL1α_1 _is consistent with recent reports on the related R29-DBL1α_1 _[49] and the responses to individual domains of the A4*var *gene [20], although this was not observed in serological surveys using an array of domains from a number of *var *genes [23,24].

VarO-IE surface reactivity correlated best with the anti-NTS-DBL1α_1 _IgG, and less strongly with the other domains. The recombinant NTS-DBL1α_1 _domain mediates rosetting, but a very small percentage of adults from Benin disrupted more than 50% of the VarO rosettes, differing in this regard from immune Senegalese adults who consistently displayed high VarO rosettes disrupting capacity [31]. None of the children sera disrupted VarO rosettes, including sera from AP children. This confirms previous observations with Senegalese asymptomatic children of the same age range, although most had VarO-IE surface-reactive antibodies [31]. The disconnection of rosette-disrupting antibodies with surface-reacting antibodies observed here in Benin has been reported in studies conducted in Kenya and Gabon with other rosette-forming parasites [50]. This suggests that if recognition of the IE-surface participates in protection against clinical malaria like anti-PfEMP1var2csa antibodies against placental malaria [8,16], other mechanisms than prevention/reversion of cytoadherence are brought about against rosette-forming parasites, including possibly complement-mediated lysis or phagocytosis of the IE. In the *Saimiri sciureus *monkey model, cytophilic antibodies targeting the IE surface and promoting IE phagocytosis of Palo Alto varO parasites were associated with protection against experimental blood stage challenge and protect animals when passively administered [33,34]. In this context, it is interesting to note that indeed a significant IgG1 and IgG3 response to each of the three recombinant varO domains could be documented in the Beninese children. It is thus possible that cytophilic antibodies to the IE surface contribute to parasite clearance in individuals that have not (yet) acquired rosette-disrupting antibodies. Both likely contribute to protection, but their acquisition may be sequential and/or depend on endemicity and transmission intensity.

SM and UM children presented similar responses in all serological assays used here. No changes in the conclusions of the analysis were brought when considering the sub-group of CM children. Based on previous studies in Gambian [32] or Gabonese children [51] such a difference might have been anticipated. The intensity of surface reaction with VarO-IE tended to be lower in the SM than in UM Beninese children studied here, but this difference did not reach significance. The high parasite density in SM children may be associated with capture of antibodies onto the parasite antigens and could account for the observed lack of differences between SM and UM children. Because of the small volume of plasma available, the isotype of surface reacting antibodies and the IgG2 and IgG4 antibodies to the recombinant domains were not measured. Therefore, the possibility exist that SM and UM children differ with regard of these isotypes. IgG3 antibodies to surface variant antigens have been reported [52], although other isotypes are produced as well [53]. In one study, IgG4 responses were reported as contributing to protection [54]. Further studies are needed to clarify this issue and evaluate the respective role of anti-surface, anti-rosetting and antibody isotype in the anti-varO response and their potential contribution to protection against severe or uncomplicated malaria.

In asymptomatic carriers, the frequency of some antibody responses decreased between 2006 and 2008, while anti-NTS-DBL1α_1 _IgG1 and anti-CIDRγ IgG did not. The level of antibodies to the recombinant antigens did not drop. This might reflect the short-lived response and/or the need of sustained asymptomatic infection documented in other settings [52,55]. The children identified as *P. falciparum *asymptomatic carriers in 2006, were parasite-free when recruited in January 2008, except for six children. It is unknown whether these parasite-free children had been and for how long, asymptomatic carriers during the 13 months elapsed between the blood samplings. The 2006-2008 period matched precisely with a large-scale distribution of long lasting insecticidal nets [56] to vulnerable populations (pregnant women and children under 5 years old) in most towns and villages in the south of Benin. This scaling-up of control measures has very likely reduced transmission - a sign of this impact might be the very low rate of asymptomatic infections in the children recruited in 2008. A reduced circulation of *P. falciparum *parasites in the field, including VarO or VarO-related parasites, resulting in reducing asymptomatic carriage and its consequences on maintenance of immune responses during this period is therefore plausible.

## Conclusions

This study confirms the elevated seroprevalence of VarO in semi-immune children and immune adults previously observed in a Senegalese population [31]. It provides evidence for the production of cytophilic antibodies to individual PfEMP1 domains. Surface reacting antibodies correlated with presence of antibodies reacting with the N-terminal NTS-DBL1α_1 _domain, consistent with the stronger response detected to NTS-DBL1α_1 _compared to the other domains. The observation of much lower anti-varO responses in children with clinical malaria compared to semi-immune asymptomatic children is consistent with an association of anti-VarO antibodies with protection against clinical malaria. Interestingly semi-immune children did not have detectable levels of antibodies capable of disrupting varO rosettes. This raises the intriguing possibility that cytophilic antibodies contribute to protection against clinical malaria by promoting opsonization of the IE, thereby reducing overall the parasite load.

## Competing interests

The authors declare that they have no competing interests.

## Authors' contributions

IVW, FMN and OMP designed the study. FMN and AG recruited the children and the adults and together with AL conducted the field work. IVM and MG performed the IE surface reactivity and rosette disrupting assays, prepared the crude extract from single variant varO cultures. AL and MG performed the ELISA assays. AJ and GB prepared the recombinant antigens. IVW, AL, AG, OMP and FMN analysed the data. IVW, AL, OMP and FMN drafted the manuscript. The final manuscript was read and approved by all authors.

## Supplementary Material

Additional file 1 **Comparison between 2006 and 2008 of prevalence rates and median values of surface-reactive, varO-IE extract-reactive and recombinant domain-reactive antibodies, among 45 children classified as asymptomatic (AP) in 2006**. *^a ^Median values (interquartile range IQ25-75), including responders only, and expressed in arbitrary units (AU). ^b ^P value of the paired χ^2 ^test. ^c ^P value of the Mann-Whitney U-test, applied to values of responders*. Click here for file
