# Supplementary material for: The humoral response to Plasmodium falciparum VarO rosetting variant and its association with protection against malaria in Beninese children
Source: Malar J. 2010 Oct 5;9:267. doi: 10.1186/1475-2875-9-267 (PMC2959068; doi:10.1186/1475-2875-9-267)
Supplement: Additional file 1 — Comparison between 2006 and 2008 of prevalence rates and median values of surface-reactive, varO-IE extract-reactive and recombinant domain-reactive antibodies, among 45 children classified as asymptomatic (AP) in 2006. a Median values (interquartile range IQ25-75), including responders only, and expressed in arbitrary units (AU). b P value of the paired χ2 test. c P value of the Mann-Whitney U-test, applied to values of responders. [file 1475-2875-9-267-S1.DOC]

| **Antigens** | **2006 (n = 45)** | |  | **2008 (n = 45)** | | ***P* ^b^** | ***P* ^c^** |
| --- | --- | --- | --- | --- | --- | --- | --- |
|  | **% of responders (95% CI)** | **Median values (AU) ^a^** |  | **% of responders (95% CI)** | **Median values (AU)** |  |  |
| **VarO-IE surface IgG** | 91 (83-99) | 59.2 (34.2-96.3) |  | 76 (63-88) | 42.1 (23.1-79.7) | 0.04 | 0.15 |
| **varO-IE extract IgG** | 100 | 85.3 (78.3-92.9) |  | 93 (86-100) | 67.9 (46.1-85.1) | 0.25 | 0.0007 |
| **NTS-DBL1α_1_: IgG** | 87 (77-97) | 87.3 (69.8-104.5) |  | 62 (48-76) | 89.4 (76.7-99.6) | 0.002 | 0.99 |
| **IgG1** | 98 (93-100) | 93.3 (82.4-98.8) |  | 93 (86-100) | 90.2 (57.2-97.6) | 0.48 | 0.17 |
| **IgG3** | 93 (86-100) | 64.8 (45.4-85.2) |  | 58 (43-72) | 46.7 (40.9-78.9) | 0.0002 | 0.15 |
| **CIDRγ: IgG** | 53 (39-68) | 62.3 (47.6-96.8) |  | 38 (24-52) | 62.0 (53.2-77.0) | 0.15 | 0.73 |
| **IgG1** | 53 (39-68) | 80.9 (64.8-97.2) |  | 36 (22-50) | 81.2 (60.2-84.4) | 0.08 | 0.52 |
| **IgG3** | 49 (34-63) | 64.9 (51.3-81.5) |  | 27 (14-40) | 74.7 (53.7-90.9) | 0.02 | 0.23 |
| **DBL2βC2: IgG** | 71 (58-84) | 65.3 (46.5-77.8) |  | 38 (24-52) | 51.0 (45.5-78.0) | 0.0007 | 0.75 |
| **IgG1** | 42 (28-57) | 82.7 (77.9-100.4) |  | 18 (7-29) | 90.8 (81.9-100.0) | 0.005 | 0.59 |
| **IgG3** | 58 (43-72) | 65.0 (50.7-78.0) |  | 18 (7-29) | 47.5 (45.5-71.9) | 0.0001 | 0.37 |
